# Optimizing dry milling of stir-cast and heat-treated AZ80 magnesium alloy using multiple criteria optimization technique: an experimental study

**DOI:** 10.1038/s41598-024-77174-3

**Published:** 2024-10-28

**Authors:** M. Tamil Selvan, M. Somasundaram, A. Raja Annamalai

**Affiliations:** 1 School of Mechanical Engineering, Vellore Institute of Technology, Vellore, TamilNadu 632014 India; 2 Centre for Innovative Manufacturing Research, Vellore Institute of Technology, Vellore, TamilNadu 632014 India

**Keywords:** AZ80 magnesium alloy, Dry milling, Response surface methodology, Heat treatment, Energy science and technology, Engineering

## Abstract

The primary aspects of this research are to evaluate surface roughness, cutting force, and material removal rate and optimize it with dry milling process parameters for heat-treated and stir-cast AZ80 magnesium alloy. Multiple methodologies are utilized in the research, which includes the Integration of design of experiments—response surface methodology for experimental design with the technique for order of preference by similarity to ideal solution for multi-criteria optimization. In order to evaluate the effect of process parameters on the response, the experimental design manipulates the depth of cut, feed rate, and cutting speed in a systematic manner. An evaluation of the machined surface’s quality is conducted via surface roughness measurements. Likewise, insights into the forces exerted during milling can be obtained through continuous monitoring of cutting forces. The calculation of material removal rate is predicated on weight reduction. The interaction between the depth of cut and feed rate has a significant impact on the critical-to-quality characteristics of the alloy, which has contribution percentage greater than 25%. This finding validates that despite the heat-treated alloy having a similar composition to the as-cast alloy (where the closeness coefficient is 0.9843), the optimal process parameters of the former are not applicable to the latter. Nevertheless, the technique used to prepare the specimen has no bearing on the material removal rate, which is a process parameter-specific effect.

## Introduction

The potential of magnesium (Mg) alloys in the engineering of lightweight structures has garnered increased interest, especially in light of the worldwide energy crisis. These materials present numerous benefits when implemented in structural contexts. These materials are distinguished by their advantageous combination of characteristics, which includes a lower density of 1.74 gm/cm^3^ and weights approximately 35% and 78% lighter than iron (Fe) and aluminum (Al), respectively. In addition to these attributes, magnesium alloys possess favorable suspension capacities, castability, weldability, machinability, and recyclability. In the twenty-first century, magnesium alloys are considered “the green material” with the most application potential due to their light weight, high specific strength and impact resistance, effective electromagnetic shielding, simple recycling, and thermal conductivity^1^. However, its cold workability at room temperature is significantly restricted due to its hexagonal close-packed (HCP) crystal structure, which impedes its effectiveness as a wrought material. The extensive range of benefits associated with magnesium (Mg) alloy milling renders it a critical process in numerous sectors. By achieving this weight reduction without compromising the alloy’s exceptional specific strength, this method has become an essential component in sectors where weight is critical, such as aerospace and automotive^2^. The automotive and aerospace industries have been identified as the primary users of the casting process to produce components with improved mechanical properties using light metals^3^. The evaluation of the impacts of casting process parameters has been primarily concentrated on composites of magnesium alloys^3^. In addition, it guarantees accuracy by providing components with precise measurements and stringent tolerances. It is of utmost importance in industries where precision is core. Furthermore, superior surface finishes can be achieved through milling, which eliminates the need for additional finishing stages. Among the diverse range of magnesium alloys that are commercially accessible, AZ alloys, which belong to the Mg–Al–Zn ternary system, have been extensively utilized in industrial contexts. Illustratively, Mg AZ80 is a prominent constituent of this category, habitually comprising 8.0 wt% Al, 0.5% Zn, and a minute quantity of Mn employed as an alloying element. This Mg alloy of moderate strength is favoured for numerous industrial applications due to its exceptional forging capabilities and resistance to corrosion^4,5^. Magnesium alloy can have its mechanical properties improved via extrusion, forging, and rolling. In order to further enhance the mechanical properties of structural materials to meet specific requirements, subsequent thermal treatment may be applied. AZ80 alloy, which is distinguished by the substantial addition of zinc and aluminum, is amenable to thermal treatment for the purpose of reinforcement. In recent times, a range of thermal treatment methodologies have been devised with the intention of augmenting the mechanical characteristics of deformed magnesium alloy, including T6 and T4 treatment. A systematic investigation was conducted to examine the impact of solution and ageing thermal treatment on the microstructure and mechanical properties of as-cast AZ80 magnesium alloy. Ultimately, an optimal solution and two-stage ageing heat treatment process were determined^6^. Recently, the cyrogenic milling behaviour of AZ91 Mg alloy was analyzed^7^. The machinability of AZ80 based composites were studied and the process parameters were optimized based on Teaching-Learning based algorithm (TLBO)^8^. AZ80, a high-strength magnesium alloy consisting predominantly of magnesium (Mg), aluminum (Al), and zinc (Zn), demonstrates additional advantageous characteristics, including reduced hardness and enhanced ductility. The exceptional machinability of this alloy facilitates the fabrication of complex and accurate parts, thereby substantially diminishing manufacturing expenses and time. While AZ80 can be utilized for effective material removal through milling, this process may result in tool fatigue and surface damage. As a process of rapid material removal, milling provides an ideal environment in which to investigate the Material Removal Rate (MRR) of AZ80 magnesium alloy. By providing operators with precise control over the machining process, it is possible to attain the desired surface finishes and dimensional accuracy. Milling is a suitable process for AZ80 magnesium alloy due to its favorable surface finishes, high material removal rates, and precision. By generating attribute evaluations, the Technique for Order of Preference by Similarity to Ideal Solution (TOPSIS) is of critical importance. Response Surface Methodology (RSM) is an effective approach for approximating the functional relationship between independent variables and the response variable in a practical manner. RSM is utilized to determine the optimal process parameter combination that yields the highest or lowest response value^9^. The research on the tribological characteristics of AZ80 indicates that the wear resistance of T6 heat-treated alloy is superior. At both room temperature and elevated temperatures, this alloy undergoes delamination and corrosive (oxidation) wear mechanisms^10^. In the meantime, the rate at which the tool advances across the workpiece is determined by the feed rate, which has a direct impact on the rates at which material is removed and the attrition of the tool. It is necessary to conduct extensive experimentation and analysis in order to tailor these parameters to the specific needs of each milling application^11^. Exploration and optimization of the dry milling process parameters for heat-treated AZ80 magnesium alloy is the purpose of this research paper. The objective of the study was to comprehensively examine the impact of T4 and T6 heat treatment and milling process parameters on critical response variables by employing response surface methodology. The study specifically investigates the effects of these variables on key performance indicators, including cutting force, material removal rate, and surface roughness. The study’s objective is to offer a thorough comprehension of the complex correlation between heat treatment conditions and milling process parameters. By doing so, it will provide valuable insights that can be utilized to optimize the end-milling of AZ80 magnesium alloy. This, in turn, will enable the achievement of improved surface quality and material removal efficiency while requiring minimal cutting force. An investigation into the impact of the heat treatment procedure on quality attributes was also provided, along with a novel method for examining the relationship between cutting speed, feed rate, and depth of cut and response variables.

## Materials and methods

### Sample preparation and experimentation

**Fig. 1:**
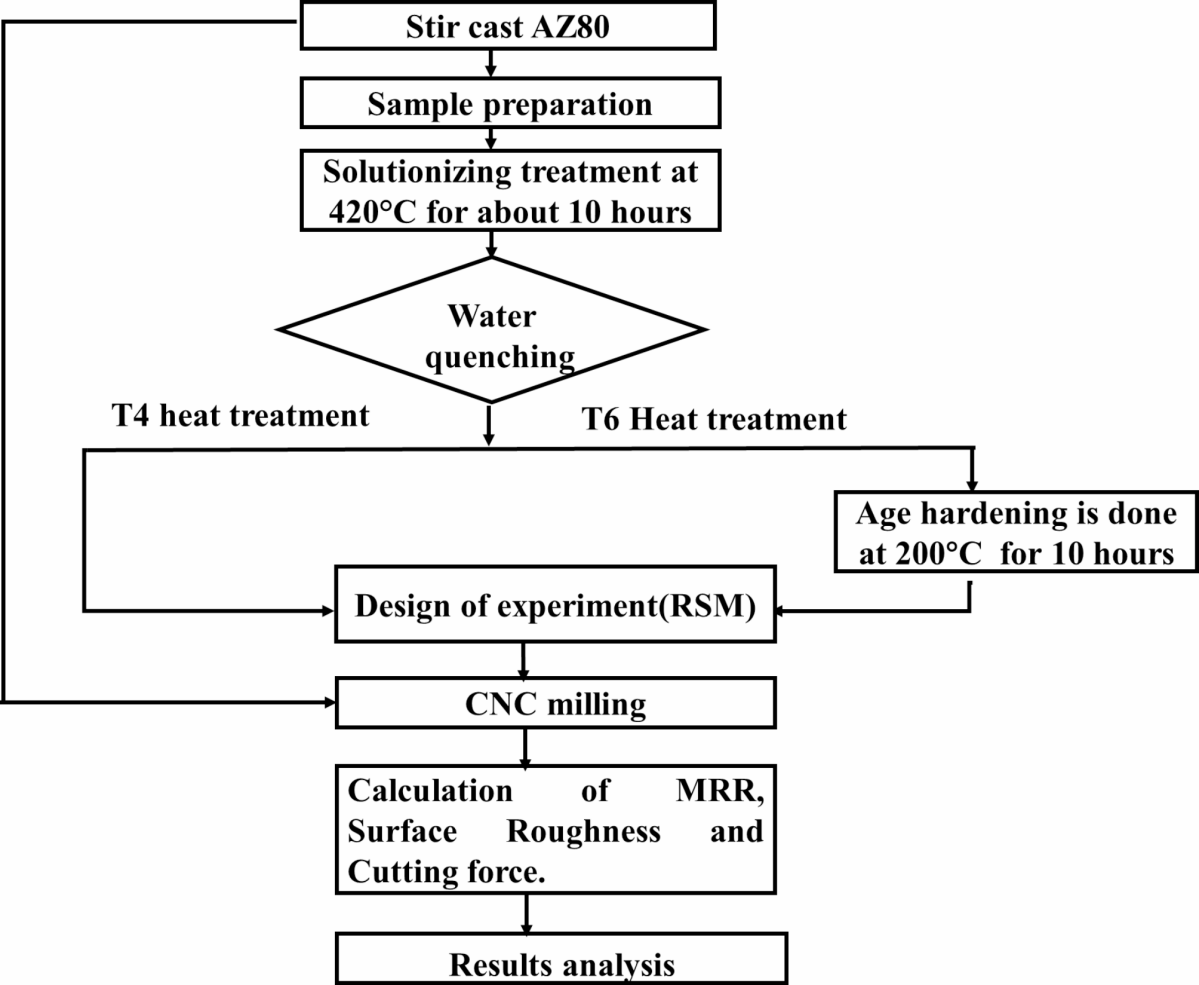
Schematic representation of the methodology of this study.

An AZ80 Mg alloy, fabricated via stir-casting techniques, serves as the specimen under investigation. The elemental composition of the prepared alloy is estimated using EDS analysis, which is Mg-8.2Al-0.61Zn-0.2Si-0.09Mn-0.07Cu. This study utilizes three separate sets of AZ80 samples: one in its as-cast condition and two after T4 and T6 heat treatments. The sample heat treated at a solutionizing temperature of 420 °C for 10 h in a muffle furnace and water quenching is termed as T4 heat treatment process. The age-hardening process utilized to treat the solutionized specimen for an additional 10 h at 200 °C is known as the T6 heat treatment process. The microstructure of the prepared alloy was analysed by Field Emission Scanning Electron Microscopy (FE-SEM). The comprehensive methodology of the research is illustrated in Fig. 1. The critical input parameters for dry milling AZ80 Magnesium Alloy are as follows: spindle speed (rpm), feed rate (mm/min), and depth of cut (mm). The upper and lower limits for each of these parameters are specified in Table 1.

**Table 1:** Process parameters of dry milling of AZ80 mg alloy.

Process parameters	Level 1 (− 1)	Level 2 (0)	Level 3 (1)
Spindle speed (S) (rpm)	500	1000	1500
Feed rate (f) (mm/min)	50	100	150
Depth of cut (d) (mm)	0.5	1	1.5

**Fig. 2:**
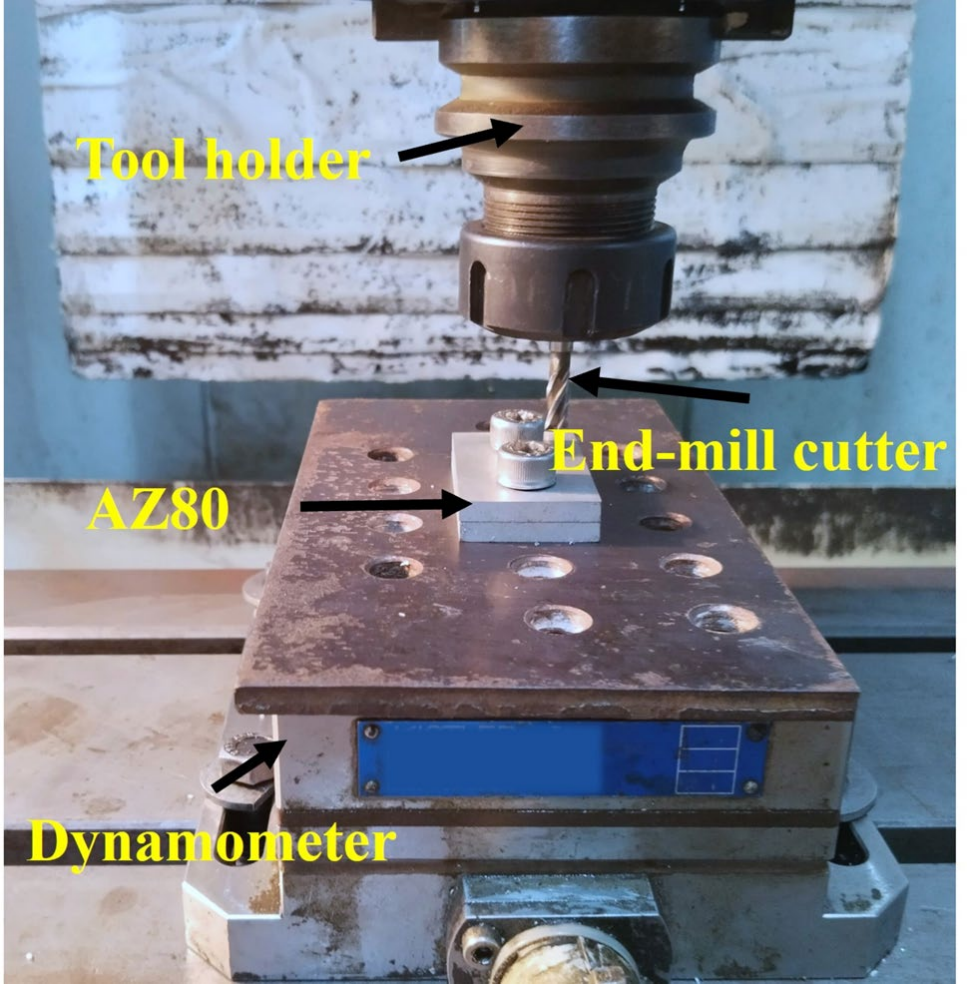
Experimental setup of end milling of AZ80 Mg alloy.

The study utilized an experimental design that was founded upon the fundamental principles of response surface methodology (RSM). The RSM method is extensively recognized and implemented in experimental design due to its ability to evaluate the influence of multiple factors and their interactions on one or more response variables. By employing RSM in this study, the effect of varying cutting speed, feed rate, and depth of cut on the surface quality (as measured by R_a_, R_z_, S_a_, S_z_), Material Removal Rate (MRR), and Resultant Force (F_r_) will be investigated^12,13^. The DoE for the Dry Milling of AZ80 Magnesium alloy is given in Table 2. Dry milling was performed on AZ80 utilizing a CNC vertical milling machine. It measured 50 mm by 30 mm by 5 mm in size. The machining was accomplished utilizing a high-speed steel (HSS) end mill tool with a diameter of 6 mm. With the intention of quantifying the cutting force, the specimen was positioned above the dynamometer (9255 B, Kistler – Singapore). The experimental setup is shown in Fig. 2. The calculation of the material removal rate (MRR) for dry milling AZ80 involves dividing the discrepancy in specimen weight between the conditions before and after machining by the total machining time. The unit of expression is gm/min. The optical profilometry system was employed to determine the surface roughness of the machined specimens (Surtronic 25, Talysurf CCI, Taylor Hobson) with a 20X objective lens at 0.8 mm^2^.

### Multi-criteria optimization

The Technique for Order of Preference by Similarity to Ideal Solution (TOPSIS) methodology is a widely used multi-criteria decision-making (MCDM) approach that helps in identifying the best option from a set of alternatives based on their relative closeness to an ideal solution. The core idea of TOPSIS is to determine the geometric distance of each alternative from both the ideal (best possible) and the nadir (worst possible) solutions. The alternative closest to the ideal solution and farthest from the nadir solution is considered the best. The TOPSIS process involves several steps: constructing the decision matrix by listing all alternatives and criteria, normalizing the decision matrix to convert criteria values into a comparable scale, applying weights to the normalized criteria based on their importance, identifying the ideal and nadir solutions by selecting the best and worst values for each criterion, computing the Euclidean distance of each alternative from the ideal and nadir solutions, determining the relative closeness of each alternative to the ideal solution, and finally ranking the alternatives based on their relative closeness, with the highest-ranked alternative being the optimal choice. The significance of TOPSIS over other optimization methods lies in its simplicity, efficiency, and ability to handle both qualitative and quantitative data. Unlike other MCDM methods such as Analytical Hierarchy Process (AHP) or Elimination and Choice Expressing Reality (ELECTRE), TOPSIS directly accounts for the relative closeness to an ideal solution, making it more intuitive and easier to understand. Additionally, TOPSIS is less computationally intensive and can handle large datasets efficiently. It provides a clear and straightforward ranking of alternatives, which is particularly beneficial in practical applications where decision-makers need quick and reliable solutions.

**Fig. 3:**
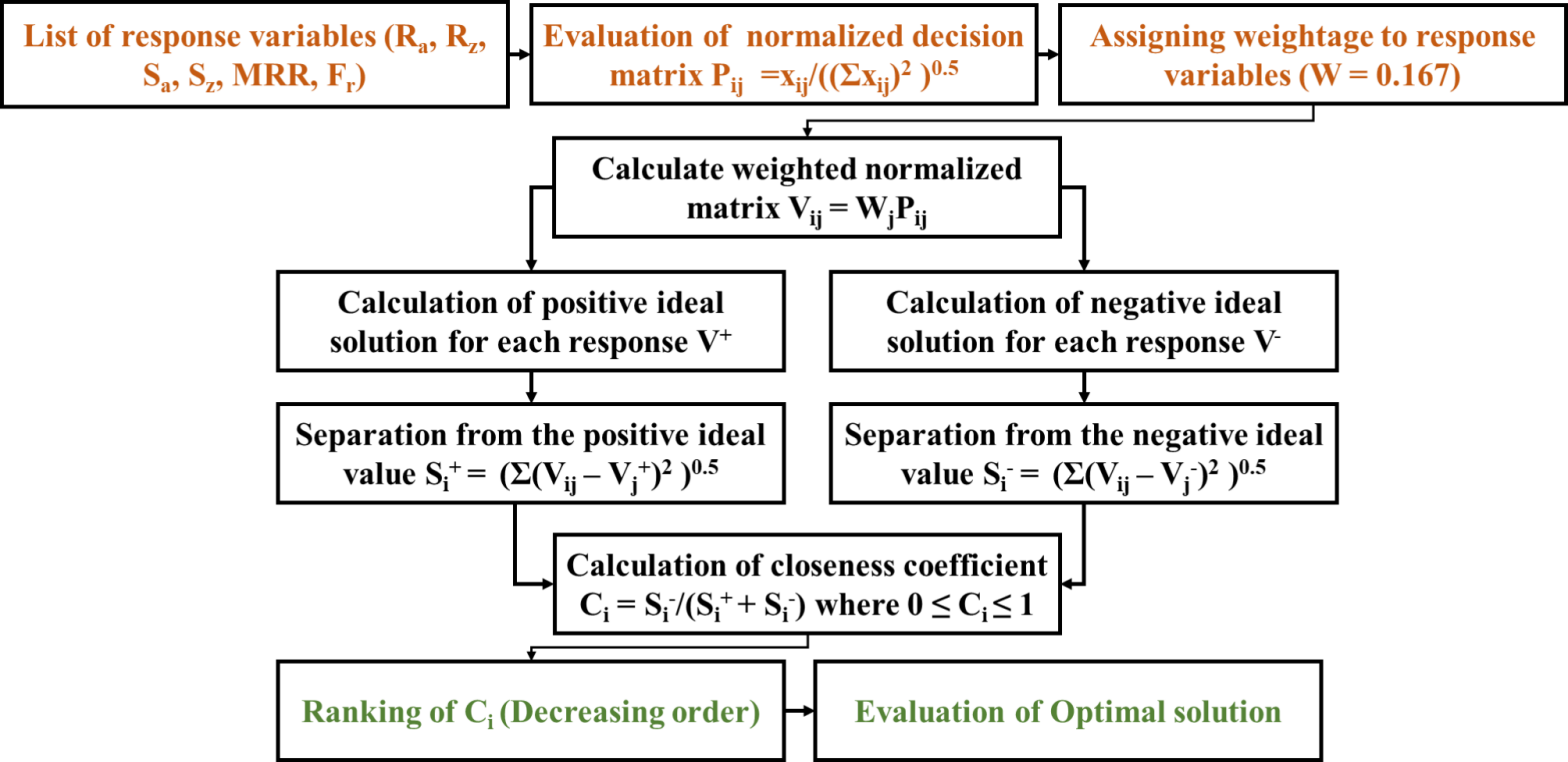
Schematic representation of TOPSIS methodology.

The preceding steps of the TOPSIS methodology are illustrated in Fig. 3. In this study, the TOPSIS method is utilized to resolve the results of multi-objective experiments through a singular optimization procedure. By giving equal weight to each response variable, this study attempts to achieve a maximum material removal rate (MRR) while minimizing surface irregularity and cutting force (F_r_). The ultimate determinations are reached upon achieving the utmost quantifiable term known as the closeness coefficient (C_i_).

## Results and discussions

**Fig. 4:**
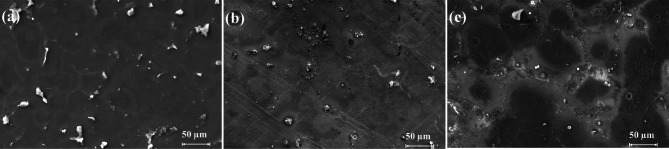
FE-SEM micrograph of (**a**) as-cast; (**b**) T4; (**c**) T6 AZ80 Mg alloy.

**Fig. 5:**
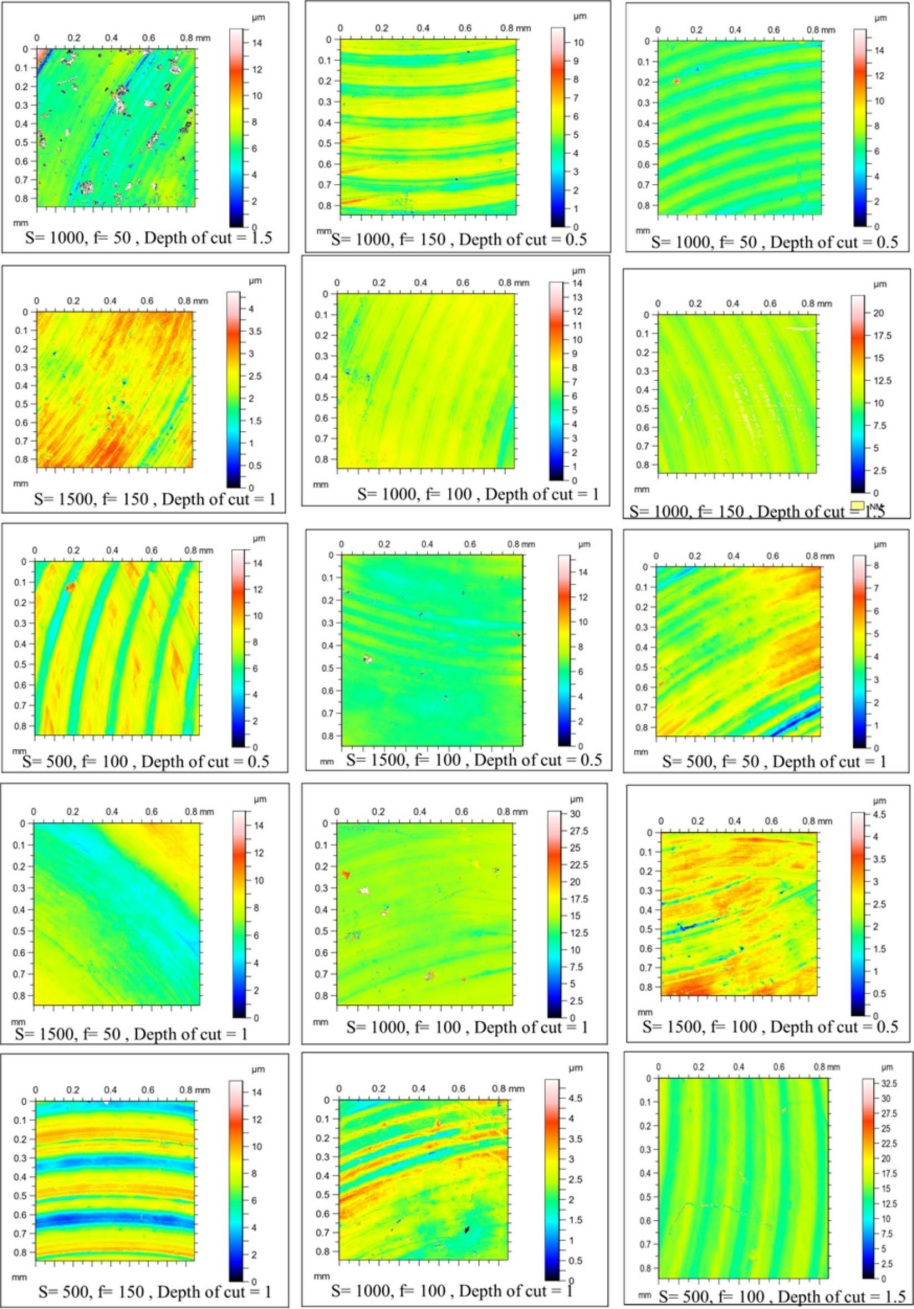
2D surface morphology of as-cast AZ80 Mg alloy after dry milling under all experimental trials.

**Fig. 6:**
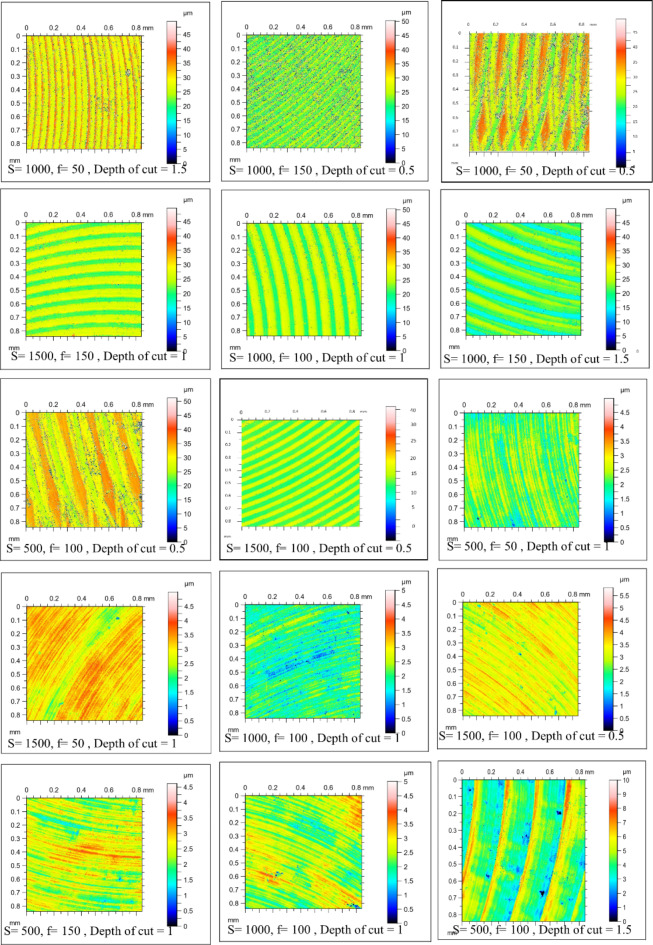
2D surface morphology of T4 heat-treated AZ80 Mg alloy after dry milling under all experimental trials.

**Fig. 7:**
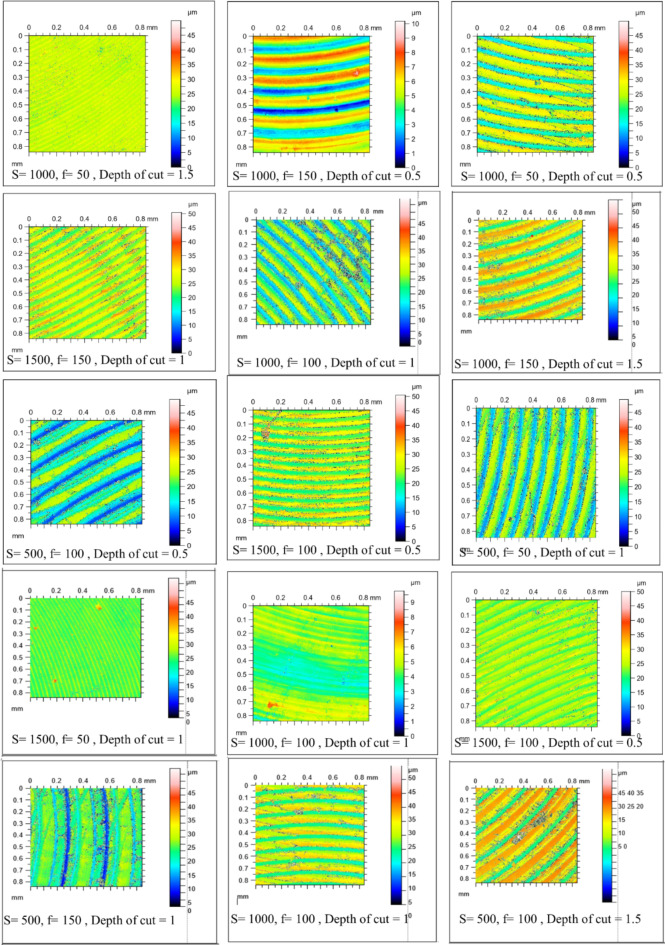
2D surface morphology of T4 heat-treated AZ80 Mg alloy after dry milling under all experimental trials.

**Fig. 8:**
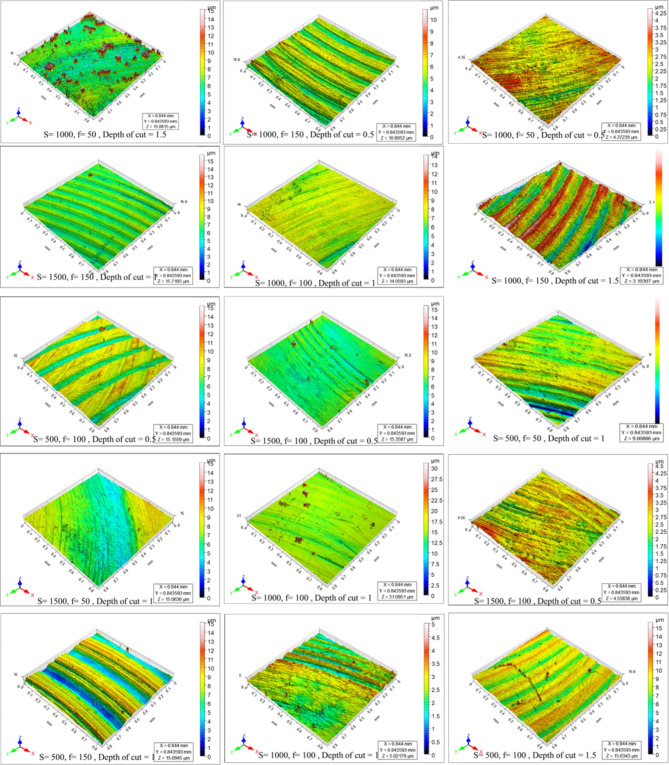
3D surface morphology of as-cast AZ80 Mg alloy after dry milling under all experimental trials.

**Fig. 9:**
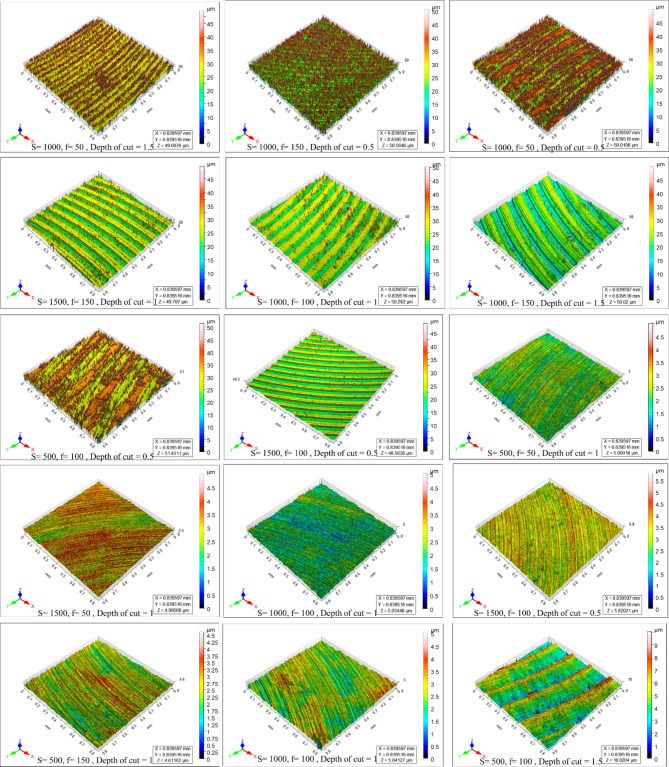
3D surface morphology of T4 heat-treated AZ80 Mg alloy after dry milling under all experimental trials.

**Fig. 10:**
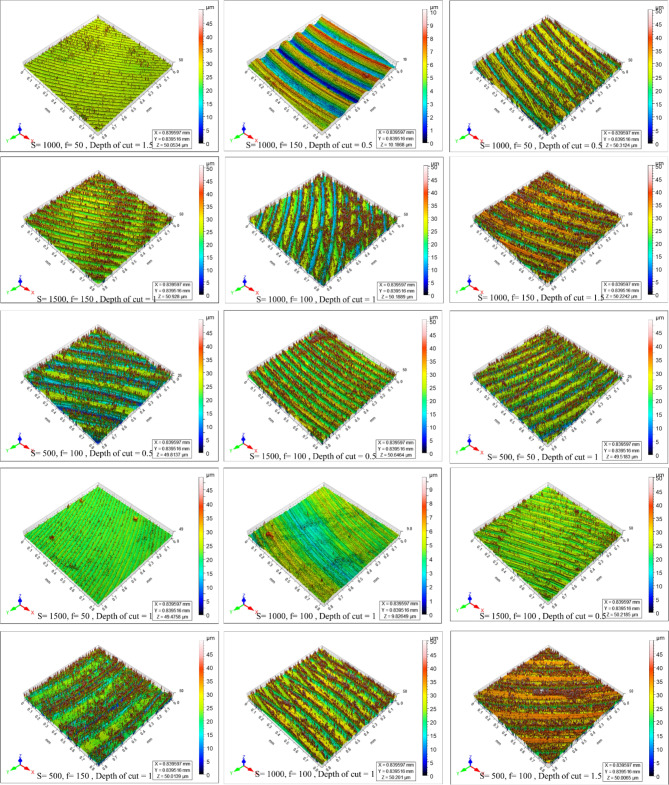
3D surface morphology of T6 heat-treated AZ80 Mg alloy after dry milling under all experimental trials.

Figure 4 shows the microstructure of as-cast, T4, and T6 AZ80 Mg alloy. Figures 5, 6, 7, 8, 9 and 10 exhibit the optical profilometry results for AZ80 Mg alloys in their as-cast, T4, and T6 states. Figures 5, 6 and 7 display the 2D surface morphology, while Figs. 8, 9 and 10 demonstrate the 3D surface morphology. Surface roughness after heat treatment is significantly higher than that of as-cast alloy, according to optical profilometry findings. As the alloy undergoes heat treatment, its strength is directly proportional to the increase in surface roughness. The tensile strength of T4 alloy improved by 14.01% and that of T6 alloy increased by 5.16% when compared with as-cast alloy, respectively, according to our prior experimental results on the mechanical properties of AZ80. Grain coarsening is also seen in the alloy that has been heat treated^14^. According to the literature, surface roughness is determined by grain size. Nevertheless, the correlation between grain size and roughness remains ambiguous^15,16^. The experimental findings of this investigation indicate that the heat-treated alloy develops a substandard surface finish due to the coarsening of granules and the increase in strength. Tables 2 and 3, and 4 present the experimental outcomes pertaining to dry milling of as-cast, T4, and T6 AZ80 alloy, respectively. Our previous study shows that “the grain size of the as-cast, T4, and T6 AZ80 are 44.43 ± 17 µm, 71.48 ± 29 µm and 72.64 ± 25 µm, respectively”^14^. The cutting force obtained is as follows: as-cast > T6 > T4. The cutting force observed in T4 AZ80 was the lowest. As-cast Mg_17_Al_12_ intermetallic phases are discernible, whereas T6 AZ80 is predominately composed of Mg_2_Al_3_ intermetallic. Conversely, a greater volume percentage of α-Mg is present in T4 AZ80, rendering it gentler in comparison to intermetallic phases. This characteristic diminishes the cutting force required to sever heat-treated alloy^14^. The cutting forces diminish as the number of grain boundaries per unit surface area decreases in conjunction with an increase in grain size^16^. The surface integrity is affected by the alloy’s strength, and the cutting force required to process the material is affected by the grain size. In all test settings, the MRR of as-cast, T4, and T6 AZ80 is similar, regardless of the specimens.

**Table 2:** Experimental results of dry milling of as-cast AZ80 mg alloy.

Trial no	Spindle speed (rpm)	Feed rate (mm/min)	Depth of cut (mm)	*R*_a_ (µm)	*R*_z_ (µm)	S_a_ (µm)	S_z_ (µm)	MRR (gm/min)	Resultant Force (*N*)
1	1000	50	1.5	0.201	1.632	1.071	15.082	0.727	182.600
2	1000	150	0.5	0.098	0.609	0.618	10.805	0.656	37.880
3	1000	50	0.5	0.084	0.460	0.251	4.372	0.229	31.480
4	1500	150	1	0.089	0.465	0.474	15.711	0.943	55.910
5	1000	100	1	0.063	0.360	0.420	14.059	0.888	25.120
6	1000	150	1.5	0.070	0.402	0.373	3.103	3.248	85.890
7	500	100	0.5	0.106	0.603	1.095	15.156	0.542	49.140
8	1500	100	1.5	0.063	0.425	0.360	15.359	1.415	181.900
9	500	50	1	0.046	0.279	0.525	9.069	0.487	118.530
10	1500	50	1	0.060	0.338	1.067	15.064	0.438	131.880
11	1000	100	1	0.065	0.398	0.487	31.095	0.823	110.060
12	1500	100	0.5	0.064	0.377	0.264	4.558	0.532	36.400
13	500	150	1	0.099	0.560	1.746	15.095	1.403	95.890
14	1000	100	1	0.044	0.268	0.371	5.022	0.968	121.710
15	500	100	1.5	0.091	0.509	0.994	15.634	1.027	154.040

**Table 3:** Experimental results of dry milling of T4 heat-treated AZ80 mg alloy.

Trial no	Spindle speed (rpm)	Feed rate (mm/min)	Depth of cut (mm)	*R*_a_ (µm)	*R*_z_ (µm)	S_a_ (µm)	S_z_ (µm)	MRR (gm/min)	Resultant Force (*N*)
1	1000	50	1.5	2.532	16.482	4.340	49.608	0.521	9.149
2	1000	150	0.5	3.810	22.526	7.095	50.011	1.179	4.926
3	1000	50	0.5	4.274	26.162	5.228	50.565	0.089	9.696
4	1500	150	1	0.535	3.607	2.864	49.707	1.260	26.567
5	1000	100	1	1.121	8.126	2.823	50.282	0.804	17.399
6	1000	150	1.5	0.670	3.914	2.768	50.020	0.664	2.865
7	500	100	0.5	2.518	14.930	4.932	51.431	0.216	10.738
8	1500	100	1.5	0.473	3.151	2.637	48.564	0.774	23.142
9	500	50	1	0.125	0.647	0.295	5.000	0.226	20.062
10	1500	50	1	0.115	0.562	0.293	4.996	0.322	24.449
11	1000	100	1	0.118	0.639	0.301	5.034	0.670	28.748
12	1500	100	0.5	0.103	0.468	0.264	5.820	0.356	18.709
13	500	150	1	0.084	0.406	0.330	4.612	0.404	27.165
14	1000	100	1	0.119	0.517	0.367	5.041	0.645	26.485
15	500	100	1.5	0.093	0.474	0.939	10.020	1.197	47.187

**Table 4:** Experimental results of dry milling of T6 heat-treated AZ80 mg alloy.

Trial no	Spindle speed (rpm)	Feed rate (mm/min)	Depth of cut (mm)	*R*_a_ (µm)	*R*_z_ (µm)	S_a_ (µm)	S_z_ (µm)	MRR (gm/min)	Resultant Force (*N*)
1	1000	50	1.5	0.948	5.979	1.358	50.053	0.642	176.107
2	1000	150	0.5	0.108	0.527	1.544	10.187	1.289	96.442
3	1000	50	0.5	3.785	26.605	6.748	50.312	0.540	87.557
4	1500	150	1	1.542	10.112	4.274	50.928	0.819	15.909
5	1000	100	1	2.109	14.474	7.011	50.189	0.749	18.122
6	1000	150	1.5	1.886	12.801	6.546	50.224	1.874	37.277
7	500	100	0.5	2.121	13.632	6.104	49.814	0.233	12.494
8	1500	100	1.5	2.765	15.989	5.647	50.646	1.051	25.779
9	500	50	1	2.576	15.945	6.575	49.518	0.367	21.195
10	1500	50	1	0.420	2.701	1.259	49.476	0.344	13.892
11	1000	100	1	0.100	0.517	0.565	9.826	0.532	17.003
12	1500	100	0.5	0.793	5.840	2.015	50.219	0.192	8.156
13	500	150	1	1.440	9.572	5.754	50.014	0.827	27.634
14	1000	100	1	2.714	18.059	5.878	50.201	0.557	15.645
15	500	100	1.5	3.616	20.955	7.254	50.007	1.281	23.193

The experimental outcomes presented in Tables 2, 3 and 4 are optimized in relation to the process parameters specified for each test condition and are regarded as response variables. The determination of the weighting of response variables is contingent upon the particular requirements of the research. The weights allocated to response parameters are distributed uniformly and are allotted an equal value of 0.167. The methodology is as follows: as illustrated in Fig. 3, the response variables are normalized prior to calculating the weighted normalized decision values matrix. In order to ascertain the degree of proximity to the ideal value solution, the calculations for the separation measures from both the positive ideal value solution and the negative ideal value solution are executed. The values of the closeness coefficients and the corresponding rankings of the different alternatives are presented in Table 5. The feasibility and impracticability of the trials are determined by comparing the C_i_ values of As Cast, T4 and T6 heat-treated samples (with the maximum C_i_ value being preferable). The TOPSIS plainly indicates that the as-cast, T4, and T6 alloys achieved their maximum C_i_ values of 0.9846, 0.9395, and 0.9374 at Trial No. 06, 14, and 11, respectively. Notably, the optimal process parameters for both heat-treated alloys are as follows: S = 1000 rpm; f = 100 mm/min; and d = 1.0 mm, as detailed in Table 6. The thermal treatment process exerts a substantial influence on various characteristics of a material, including grain size, phases, and tensile strength^17,18^. Furthermore, under all test conditions, Trial 08 exhibits undesirable responses: S = 1500 rpm, f = 100 mm/min, and d = 1.5 mm.

**Table 5:** Closeness coefficient values and ranking of alternatives. (as cast, T4, T6)

Sample	As cast		T4		T6	
Trial No	C_i_	Rank	C_i_	Rank	C_i_	Rank
1	0.9398	14	0.8733	11	0.8645	13
2	0.9634	7	0.8392	13	0.9324	2
3	0.9626	8	0.8241	14	0.8552	14
4	0.9663	5	0.9147	8	0.9197	3
5	0.9686	3	0.9101	10	0.9002	8
6	0.9846	1	0.9204	7	0.9179	5
7	0.9584	11	0.8645	12	0.8911	9
8	0.0524	15	0.1157	15	0.0301	15
9	0.9640	6	0.9282	6	0.8874	11
10	0.9584	10	0.9285	5	0.9192	4
11	0.9580	13	0.9370	2	0.9374	1
12	0.9668	4	0.9360	3	0.9106	7
13	0.9582	12	0.9289	4	0.9155	6
14	0.9695	2	0.9395	1	0.8906	10
15	0.9605	9	0.9123	9	0.8858	12

**Table 6:** Optimal process parameters to mill as-cast, T4, and T6 AZ80 mg alloy under test conditions.

Sample	Spindle speed	Feed rate	Depth of cut	C_i_
As cast	1000	150	1.5	0.9846
T4	1000	100	1.0	0.9395
T6	1000	100	1.0	0.9374

**Fig. 11:**
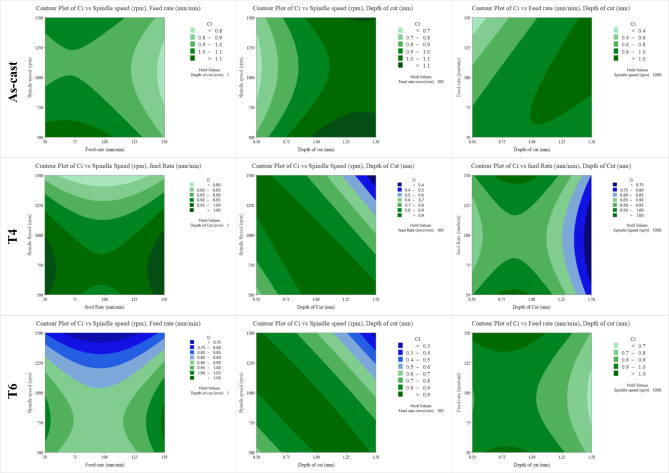
Contour plot of closeness coefficient of as-cast, T4, and T6 heat-treated AZ80 Mg alloy.

The contour plots of feed rate versus spindle speed, depth of cut versus spindle speed, and depth of cut versus spindle speed on C_i_ of as-cast, T4, and T6 AZ80 alloy are displayed in Fig. 11. The contour plot is a valuable tool for determining the impact of distinct process parameters on the response C_i_ over a specified range of process parameters. When working with as-cast AZ80, the minimum speed, maximum depth of cut, and minimum feed rate are all desirable. Lower feed rates resulted in improved surface roughness due to the reduced tool deflection and minimized vibration, which contribute to a more stable cutting process and finer surface finish. - Optimal depth of cut was found to improve surface roughness by balancing the cutting forces and minimizing tool wear, thereby maintaining a consistent and smooth cutting action. In contrast, T4 and T6 exhibit comparable patterns in the contour plot. For a consistent depth of cut and spindle speed, the optimal feed rates are 50–75 mm/min and 125–150 mm/min, respectively. It is desirable to have the highest depth of cut at the lowest spindle speed with a constant feed rate, and, conversely, for both T4 and T6 heat-treated alloys. Although the MRR increases regardless of feed rate, the rate of increase is significantly greater with a higher feed rate^19,20^.

As shown in Table 7, the ANOVA results offer a comprehensive understanding of the significance of machining parameters and their interactions in determining Ci under the three conditions of AZ80 Magnesium alloy: as-cast, T4, and T6. The ANOVA analysis indicates that the model is statistically significant in the as-cast condition, accounting for 76.19% of the total variation (C_i_). Within the realm of linear effects, depth of cut and feed rate each make significant contributions of 14.04% and 10.21%. Analogous to the current investigation, the examination of high-speed milling of magnesium alloy revealed that the depth of cut is a crucial determinant, given that an increased depth of cut leads to compromised surface quality as a consequence of vibration^21^. It is worth mentioning that the statistical analysis reveals that the interaction between feed rate and depth of incision accounts for 6.78% of the variance observed in the closeness coefficient. This emphasizes the significance of taking into account the cumulative impact of these variables. The model maintains its significance under the T4 condition, accounting for 72.23% of the overall variation. Significant factors that contribute 13.84% and 11.17%, respectively, to the closeness coefficient are depth of cut and spindle speed. A prior investigation concerning the machining of magnesium alloy demonstrated that the depth of cut is an inconsequential factor when d = 0.3, 0.4, and 0.5 mm in the pursuit of optimizing surface roughness. Additionally, the study validated the importance of feed rate^22^. It is deemed invalid when d ranges from 0.5 to 1.5 mm and when multiple responses are being optimized.

Likewise, the model maintains its significance in the T6 condition, accounting for 72.87% of the overall variation. Spindle speed and depth of cut remain influential factors, contributing 11.40% and 8.61% to the closeness coefficient, respectively. While the statistical significance of individual interaction effects is low, the combined effect underscores the potential influence that these effects may have on heat-treated alloys T4 and T6. The interaction between feed rate and depth of cut has a significant impact on the Ci under all test conditions; the Ci values for as-cast, T4, and T6 alloys are 25.60%, 31.46%, and 26.70%, respectively. In contrast to spindle speed, it verifies the effect of feed rate and depth of cut on quality-critical characteristics. On end milling, however, the as-cast AZ80 exhibits a distinct behavior from the alloy that has been heat-treated. The influence of spindle speed is substantial in the case of T4 and T6 alloy, but this is not the case with as-cast alloy.

**Table 7:** Contribution percentage of process parameters on closeness coefficient (ANOVA).

Source	DF	Contribution percentage (%)		
		As-cast	T4	T6
Spindle speed (rpm)	1	0.10	11.40	11.17
Feed rate (mm/min)	1	10.21	0.05	0.44
Depth of cut (mm)	1	14.04	8.61	13.84
Spindle speed (rpm)*Spindle speed (rpm)	1	11.84	3.59	5.72
Feed rate (mm/min)*Feed rate (mm/min)	1	6.78	4.75	7.04
Depth of cut (mm)*Depth of cut (mm)	1	7.35	12.31	7.91
Spindle speed (rpm)*Feed rate (mm/min)	1	0.27	0.01	0.03
Feed rate (mm/min)*Depth of cut (mm)	1	25.60	31.46	26.70
Error	6	23.81	0.04	0.02

## Conclusion

- The study examined dry milling of AZ80 under various conditions, including as-cast, T4, and T6 heat treatment. To optimize multiple responses, the study utilized the TOPSIS method and conducted experiments based on the RSM-Box-Behnken method. Optimizing multi-criteria issues is facilitated by the hybrid analysis (RSM – TOPSIS – ANOVA).
- The experimental findings indicate that the strength of the alloy influences the surface integrity of the material, while the cutting force needed to manufacture the material is determined by the grain size. Maximum spindle speed and depth of cut are undesirable under all test conditions when the feed rate remains constant.
- The following parameters must be met in order to achieve the desired surface quality while minimizing cutting force and maximizing MRR: S = 1000 rpm, f = 150 mm/min, d = 1.5 mm for as-cast; S = 1000 rpm, f = 100 mm/min, d = 1.0 mm for T4 and T6.
- The interaction between depth of cut and feed rate has a significant impact on critical-to-quality characteristics of the alloy, according to the ANOVA results. Under the provided experimental conditions, the impact of cutting speed was observed to be comparatively insignificant in this particular condition.
- The experimental investigation of dry milling of AZ80 Mg alloy revealed that under comparable milling conditions, the as-cast and heat-treated (T4 and T6) specimens exhibit distinct properties. Despite having a similar alloy composition, it verifies that the heat-treated alloy cannot be processed using the optimal process parameters of the as-cast alloy. However, the method of specimen preparation has no bearing on the material removal rate, which is a process parameter-specific characteristic.

## Data Availability

The datasets used and/or analysed during the current study available from the corresponding author on reasonable request.

## Abbreviation

d: Depth of cut (mm)
DoE: Design of experiments
f: Feed rate (mm/min)
FE-SEM: Field emission scanning electron microscopy
F_r_: Resultant force (N)
HCP: Hexagonal close-packed crystal structure
MRR: Material removal rate (gm/min)
R_a_: Arithmetic average surface roughness (µm)
RSM: Response surface methodology
R_z_: Maximum height of surface roughness (µm)
S: Spindle speed (rpm)
S_a_: Arithmetic mean height (3D surface roughness) (µm)
S_z_: Maximum height of 3D Surface Roughness (µm)
T4: ASM heat treatment process-solutionizing
T6: ASM heat treatment process-solutionizing + age hardening
TOPSIS: Technique for order preference by similarity to ideal solution
